# Development and Validation of a Nomogram to Predict Depression Risk in Patients with Cardiovascular Disease

**DOI:** 10.3390/healthcare13111287

**Published:** 2025-05-29

**Authors:** Zhao Li, Yu Zhao, Hyunsik Kang

**Affiliations:** College of Sport Science, Sungkyunkwan University, Suwon 16419, Republic of Korea; zhaol@skku.edu (Z.L.); zhaoyu0809@skku.edu (Y.Z.)

**Keywords:** Cardiovascular disease, depression, risk assessment, feature selection, nomogram

## Abstract

**Background/Objectives:** Approximately one-third of patients with cardiovascular disease (CVD) experience depression. This study aimed to develop and validate a nomogram for assessing the risk of depression in patients with CVD. **Methods**: In a cross-sectional study design, we analyzed data obtained from 6702 patients with CVD who participated in the 2007–2018 National Health and Nutrition Examination Survey. The dataset was randomly split into training and validation cohorts at a 0.75 to 0.25 ratio. Univariate and multivariate logistic regression analyses were applied to the training cohort to identify predictors for a web-based dynamic nomogram, which was then validated in the validation cohort. **Results**: Blood Cd concentration, sedentary time, eosinophil count, marital status, work limitations, sleep disorders, asthma, stomach or intestinal illness, confusion or memory problems, ethnicity, and cotinine were identified as risk factors for depression in patients with CVD, and these 11 risk factors were incorporated into the nomogram. The area under the curve (AUC) of the nomogram was 0.852 (95% CI: 0.842–0.862) in the training cohort, with a sensitivity of 83.28% and specificity of 72.95%. The AUC was 0.856 (95% CI: 0.838–0.872) in the validation cohort, with a sensitivity of 79.14% and a specificity of 76.65%. The C-index of the nomogram was 0.852 in the training cohort, with a mean absolute error of 0.012 based on 1000 bootstrap replicates. The C-index of the nomogram model was 0.863 in the validation cohort, with a mean absolute error of 0.017. **Conclusions**: Our nomogram model demonstrates potential clinical utility for the early screening of depression risk in patients with CVD.

## 1. Introduction

In its 2019 statistics, the World Health Organization (WHO) estimated that cardiovascular disease (CVD) accounted for approximately 32% of all-cause mortality worldwide, making it a leading cause of premature death, especially in low- and middle-income countries (https://www.who.int/data/gho/data/indicators/indicator-details/GHO/estimated-population-based-prevalence-of-depression/, accessed on 10 September 2024). Although the exact underlying mechanism(s) has yet to be determined, multiple factors, including family history, ethnicity, hypertension, smoking, high cholesterol, diabetes, physical inactivity, stress, and loneliness/social isolation, have been linked to CVD etiology [1].

Depression is a severe mental illness that impairs overall well-being, resulting in disability [2] and suicidal thoughts [3]. Approximately 280 million people worldwide or 3.8% of the total population, suffer from depression (https://www.who.int/data/gho, accessed on 10 September 2024). In addition to traditional risk factors, depression has been established as an important risk factor for CVD morbidity and mortality. People with depression exhibit a higher risk of CVD morbidity and mortality than those without depression. Concurrently, patients with CVD are more predisposed to depression than the general population [4,5], implying the coexistence of the two health conditions.

Depression affects various CVD patients, including those with coronary artery disease [6], chronic heart failure [7], angina pectoris [8], and stroke [9]. A benchmarking study of ischemic heart disease in Australia showed that up to 40% of patients experienced depression [10]. In a hospital-based study, nearly 66% of patients developed depression after being hospitalized due to cardiac disease or acute myocardial infarction [11]. Depression affects the effectiveness of rehabilitation and quality of life in patients with CVD, such that the risk of adverse events and mortality increases [12]. The association between depression and CVD has been reviewed and summarized in recent meta-analyses of prospective cohort studies [4]. Collectively, the findings of previous studies imply a pathological connection between these two health conditions [13].

Depression has a multifaceted etiology, with social, psychological, behavioral, and biological risk factors acting separately or concurrently [14]. Social risk factors include poor socio-demographics [15], loneliness and/or social isolation [16], ongoing difficulties [17], physical disabilities [18], and poor work performance [19]. Environmental factors include neurotoxic substances like cadmium, lead, mercury, and arsenic [20]. These risk factors for depression are also linked to the biological and behavioral risk factors that contribute to the development of CVD. In contrast, adopting a healthy lifestyle, such as physical activity, avoiding prolonged sitting, and a healthy diet, has antidepressant effects, lowers the risk of CVD, reduces CVD recurrence, and facilitates rehabilitation to a normal lifestyle [21]. Taken together, the findings from previous studies highlight the need for a multifaceted approach to assessing depression risk in patients with CVD because they may share risk factors [22].

Constructing a multivariate regression analysis-based nomogram may facilitate screening for depression risk among patients with CVD, enabling early detection, intervention, and rehabilitation. Unfortunately, screening for post-CVD depression risk is often ignored or insufficiently addressed, as most patients do not receive appropriate care and/or treatment [5]. This study aimed to develop and validate a nomogram model to screen for the risk of depression in patients with CVD.

## 2. Materials and Methods

### 2.1. Study Design

The National Center for Health Statistics (NCHS) of the Centers for Disease Control and Prevention (CDC) conducts the National Health and Nutrition Examination Survey (NHANES) on an annual basis to assess the health and nutritional status of adults and children in the United States through personal interviews and physical examinations. The NHANES uses a complex, multistage probability sampling design and surveys 5000 people every two years on average. After cleaning the data and removing missing samples, we selected six NHANES (2007–2018) cycles as the original dataset for analysis. The NCHS Research Ethics Review Board reviewed and approved the NHANES protocol (as well as the publicly released de-identified data). Due to the open availability of NANES, this study was exempted from the Institutional Review Board (IRB) review. Informed consent was not required for this study because it was a secondary analysis of existing datasets.

### 2.2. Participants

**Figure 1:**
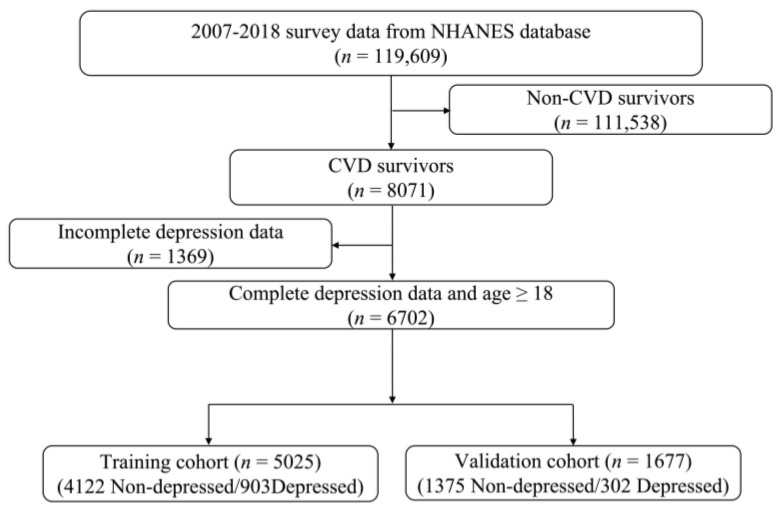
Flow chart of the study participants.

As illustrated in Figure 1, we combined six NHANES survey cycles from 2007 to 2018, with a total of 119,609 participants. We excluded participants without CVD (*n* = 111,538) and/or those without depression data (*n* = 1369). The remaining 6702 patients with CVD were included in the final data analysis. During the modeling process, 3/4 of the participants were randomly assigned to the training cohort (*n* = 5025), with the remaining 1/4 assigned to the validation cohort (*n* = 1677).

### 2.3. Data Interpolation

**Figure A1:**
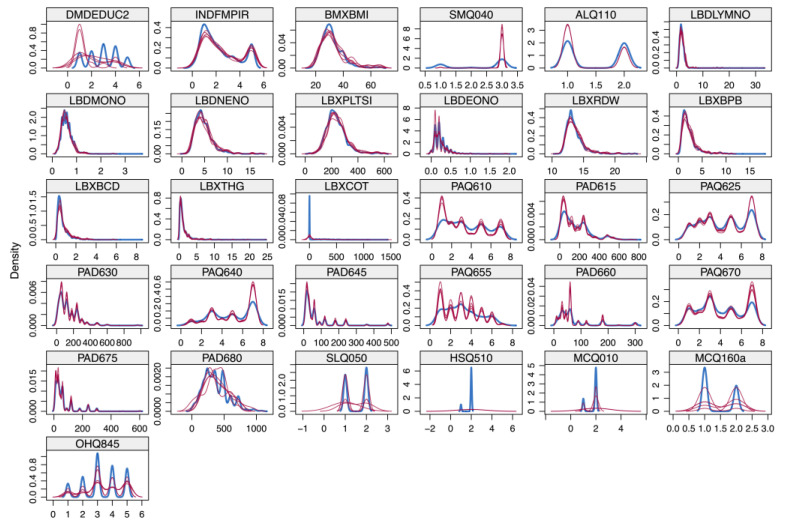
Effects of data imputation.

No imputation was performed for the primary outcomes of depression and CVD due to the absence of missing data. For missing data, we used the Jomo package for multilevel joint modeling and multiple imputations [23]. We conducted interpolation in a Bayesian framework using a survey-weighted generalized linear model [24,25]. We used the Gibbs sampling method to generate five imputed datasets after a burn-in of 1000 rounds to ensure that the imputed datasets were not affected by random events [26]. We chose the final dataset for analysis by examining model fit indicators, such as the Akaike Information Criterion (AIC) and the Bayesian Information Criterion (BIC). We did this to ensure that the selected dataset accurately represented the data distribution (Appendix B Figure A1).

The NHANES uses a stratified, multistage probability sampling design in which individuals are divided into primary sampling units and geographical strata. The Jomo package supports joint modeling using a multilevel framework, which helps account for intra-cluster correlation while retaining between-cluster variability during the imputation process [27]. This is especially important in our case to maintain the internal structure and heterogeneity of the dataset to ensure robust prediction modeling. While other survey-specific imputation approaches, such as those incorporating sampling weights, are valid, we chose a method that preserved the variability across sampling clusters. Furthermore, the Jomo package allows for the flexible imputation of datasets containing continuous and categorical variables. Given these considerations, the Jomo package is appropriate for our study and follows the best practices for handling missing data in complex survey datasets, such as the NHANES.

### 2.4. Cross-Validation: Evaluating the Predictive Model Performance

**Figure A2:**
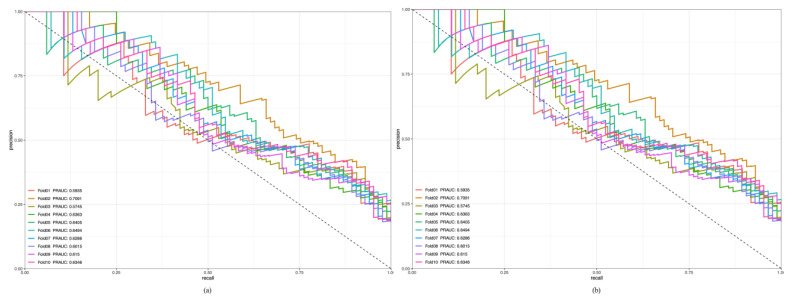
Results of 10-fold cross-validation for the training (**a**) and testing datasets (**b**).

We evaluated the performance of our predictive model using the 10-fold cross-validation method, which enhances reliability by mitigating the risk of overfitting and enhances generalization by using every data point to train and test the datasets. The dataset was randomly shuffled and partitioned into ten equal-sized folds. For each iteration, 1-fold was reserved for testing the model while the remaining folds were used for training the model. This procedure was repeated 10 times, with each fold serving as the testing set once. After the model was fitted to each training fold, predictions were generated for the corresponding validation fold. The model performance was evaluated using metrics such as accuracy, sensitivity, specificity, area under the receiver operating characteristic curve (ROCAUC), and area under the precision-recall curve (PRAUC). The cross-validation had a mean accuracy of 85.3 ± 0.5 with a PRAUC of 62.8 ± 1.17, indicating a solid predictive capability despite the observed class imbalance (Appendix B Figure A2).

### 2.5. Variables

#### 2.5.1. Definition of Cardiovascular Disease and Depression

The Medical Conditions Questionnaire (MCQ) was used to determine the presence of self-reported CVD, which was defined as having a disease(s) diagnosed by a health professional, such as congestive heart failure, coronary heart disease, angina pectoris, heart attack (myocardial infarction), or stroke. The presence of depression was determined using the nine-item Patient Health Questionnaire-9 [28]. The participants were asked about their depressive status over the previous two weeks and how troubled they were by the situation presented. Responses were rated as “not at all” (0), “several days” (1), “more than half of the time” (2), and “nearly every day” (3). The possible scores range from 0 to 27. The scores for all nine questions were combined, and a PHQ-9 score of 10 was used to determine the presence of major depression. Although a clinical diagnosis of depression requires a comprehensive evaluation that consists of physical examination, lab tests, psychiatric evaluation, and the Diagnostic and Statistical Manual of Mental Disorders, Fifth Edition (DSM-5), the use of a PHQ-9 score of ≥10 has been proven to be a sensitive and specific cut-off for identifying individuals likely to meet the criteria for depression [29]. We were interested in exploring the risk of depression in patients with CVD, helping to identify those who may benefit from early detection and intervention.

#### 2.5.2. Definition of Risk Factors

Comorbidities, health behaviors, complete blood count, blood metal toxin concentrations, and socio-demographic factors were considered potential risk factors in this study. Comorbidities included asthma, osteoarthritis, stomach or intestinal illness with vomiting and/or diarrhea, work limitations due to health conditions, mobility disorders, memory problems or confusion, sleep disorders, and use of prescribed medications. Asthma and osteoarthritis were diagnosed based on self-reports or by a physician or other professionals.

Stomach or intestinal illness was defined as a “YES” answer to the following question: Have you had a stomach or intestinal illness that caused vomiting or diarrhea within the last 30 days? Work limitations were considered present if there was a “YES” answer to the following question: Do you have any long-term physical, mental, or emotional problems or illnesses? Mobility disorders were considered present when the response to the following question was “YES”: Do you have difficulty walking without the use of special equipment due to a health condition? Memory problems or confusion were considered present based on a response of “YES” to the following question: Are you limited in any way because you have difficulty remembering or because you experience periods of confusion? Sleep disorders were considered present with a “YES” to the following question: Have you ever told a doctor or another health professional that you have difficulty sleeping? Prescription medicine use was defined as answering “YES” to the following question: Have you used or taken medication that requires a prescription in the last 30 days?

This study considered physical activity (PA), sedentary time, body mass index (BMI), smoking (smokers vs. non-smokers), heavy alcohol consumption, sleep duration [25], and oral health [30] as health behavior parameters. Daily physical activity was assessed using the International Physical Activity Questionnaire (IPAQ), which classified activities lasting ≥ 10 min into three categories: vigorous work or recreational activity (VPA), moderate work or recreational activity (MPA), and light walking or bicycle physical activity (LPA). Information on frequency (days per week) and duration (minutes per day) was collected using specific questionnaire items (e.g., PAQ605, PAQ610, PAQ615, PAQ620, PAQ625, PAQ630, PAQ635, PAQ640, PAQ645, PAQ650, PAQ665, PAQ670, PAD675). The intensity of physical activity was determined using metabolic equivalent tasks (METs), with 8.0 assigned to VPA, 4.0 to MPA, and 3.0 to LPA. The total amount of physical activity performed each week was calculated in MET-min/week by summing the VPA, MPA, and LPA MET scores, daily duration in minutes, and number of days per week. Physical activity was classified as low (less than 500 MET-min/week), moderate (500–1000 MET-min/week), or high (>1000 MET-min/week), as previously suggested [31]. BMI was categorized as underweight (<18.5 kg/m^2^), normal weight (18.5–24.9 kg/m^2^), overweight (25.5–29.9 kg/m^2^), or obese (≥30.0 kg/m^2^).

Blood samples were collected at NHANES mobile examination centers and delivered to the National Center for Environmental Health and Centers for Disease Control and Prevention. Beckman Coulter (Brea, CA, USA) MAXM instruments were used to perform a complete blood count (CBC), with CBC parameters calculated by counting, sizing, automatic diluting, and mixing samples. The systemic immune-inflammation index (SII) was calculated as the product of the platelet and neutrophil counts divided by the lymphocyte count [24]. The systemic inflammatory response index (SIRI) was calculated as the product of the monocyte and neutrophil counts divided by the lymphocyte count [32]. Eosinophil count and red cell distribution width were included. Blood concentrations of lead, cadmium, mercury, and cotinine were also measured. Detailed procedures for specimen collection and processing are available on the NHANES website (https://www.cdc.gov/nchs/nhanes/about/index.html/, accessed on 10 September 2024). 

We classified SII, SIRI, eosinophil count, red cell distribution width, and blood levels of lead, cadmium, mercury, and cotinine into quartiles (Q1–Q4) based on their population distributions. This method was chosen because these biomarkers do not have widely accepted clinical cut-off values for predicting the risk of depression. Quartile-based categorization facilitates the identification of potential dose-response relationships, reduces the influence of outliers and skewed distributions, and improves interpretability in epidemiological analyses. Age was maintained as a continuous variable because of its well-established linear relationship with depression and its importance as a covariate in statistical models. Categorizing age may result in the loss of information and decreased model performance; thus, it was left in its original form to maximize analytical accuracy.

Additionally, social and demographic factors included sex (female vs. male), ethnicity (non-Hispanic white, non-Hispanic Black, Mexican American, other Hispanic, other race), education (less than 9th grade, 9–11th grade, high school grad/GED, some college or AA degree, college graduate or above), marital status (married/living with partner, widowed/divorced/separated, never married), and family income to poverty ratio (more than 130% or less than 130%).

### 2.6. Statistical Analysis

Means and standard deviations were used to represent normally distributed data, while medians and quartiles were used for non-normally distributed data. Categorical variables were expressed as counts and percentages (%), and group comparisons were performed using the chi-square test. Pearson’s correlation analysis was performed for all variables. The variance inflation factor (VIF) was used to detect multicollinearity. Variables with the highest VIF were removed sequentially from the dataset until all variables had a VIF of <4. We used univariate logistic regression analysis to identify significant predictors (*p* < 0.05) among the extracted socio-demographic, health behavior, and clinical and laboratory parameters. The selected predictors were then entered into a multivariate logistic regression model. The association between risk factors and post-CVD depression was measured using odds ratios (OR) and 95% confidence intervals (CI). In our nomogram model, each risk factor was assigned a point based on its OR. The probability of depression risk was obtained by adding the total score of each risk factor and locating the score on a total-point scale. The accuracy of the nomogram model was assessed using an internal bootstrap method that involved 1000 repeated random samples with their replacements. The discriminative ability and predictive performance of the nomogram model were evaluated using ROC curves and calibration plots. The clinical prediction utility of the model was evaluated using decision curve analysis (DCA) and a clinical impact curve (CIC). All statistical analyses and graph visualizations were performed using R-4.3.2 for Windows [33].

## 3. Results

### 3.1. Description of CVD Patients by Depression Status

Table 1 describes CVD patients according to their depression status. Depression was present in 17.9% of the study population, with 17.9% in the training cohort (*n* = 903) and 18.0% in the validation cohort (*n* = 302). CVD patients with depression were younger, more likely to be male, Mexican American, or Hispanic, less educated, more likely to live without a partner or never to have married, more likely to be poor, obese, drink alcohol, and smoke; to have asthma, osteoarthritis, stomach or intestinal illness, work limitations, mobility disorders, confusion or memory problems, longer sleep duration, poor dental health, longer sedentary time, and lower physical activity than CVD patients without depression. Additionally, patients with CVD and depression had higher SII, blood cadmium, and cotinine levels but lower SIRI, eosinophil count, blood lead concentration, red blood cell distribution width, and Hg concentration than those without depression.

Furthermore, except for prescribed medication use, there were significant differences in all other measured parameters between the non-depressed and depressed participants in the training cohort. Except for SII, red cell distribution width, eosinophil count, prescribed medication use, and sedentary time, all other measured parameters showed significant differences between non-depressed and depressed participants in the validation cohort (Appendix A Table A1). This implies that the training and validation cohorts were somewhat heterogeneous.

### 3.2. Risk Factors for Post-CVD Depression

Table 2 shows the outcomes of the univariate and multivariate logistic regression analyses for the entire training and validation cohorts. Univariate logistic regression revealed that positive risk factors for depression were female sex, non-white ethnicity (i.e., Mexican American, Hispanic, non-Hispanic Black, and other races), living alone (i.e., not living with a partner or never married), lower family income to poverty ratio, drinking, smoking, eosinophils, blood cadmium and cotinine levels, asthma, osteoarthritis, stomach or intestinal illness, work limitations, mobility disorders, confusion or memory problems, sleep disorders, poor dental health, and sedentary time. Negative risk factors for depression included age, education, BMI, SII, SIRI, red cell distribution width, blood lead and mercury levels, sleep duration, and physical activity. Multifactorial logistic regression revealed that the positive risk factors for depression were female sex, race, never married status, lower family income to poverty ratio, drinking, SIRI, eosinophil counts, blood cadmium level, asthma, osteoarthritis, stomach or intestinal illness, work limitations, confusion or memory problems, sleep disorders, and sedentary time. Negative risk factors for depression included age, education, smoking, SII, SIRI, red blood cell distribution width, blood lead, mercury and cotinine levels, and physical activity.

### 3.3. Elimination of Multicollinearity

The existence of multicollinearity was checked by calculating the variance inflation factor (VIF). SII, SIRI, NLR, and AISI were excluded from the prediction model because their VIFs exceeded 4 (Appendix A Table A2). A web-based dynamic nomogram for predicting prediabetes risk was built using the selected features.

### 3.4. Goodness of Fit Testing

The Hosmer-Lemeshow (HL) test is a statistical tool that assesses the performance and robustness of a nomogram model by comparing the observed and expected frequencies calculated from the model. The H-L test yielded a *p*-value of 0.6397, suggesting a good fit for the nomogram prediction model.

### 3.5. Construction of a Risk Prediction Nomogram for Depression

**Figure 2:**
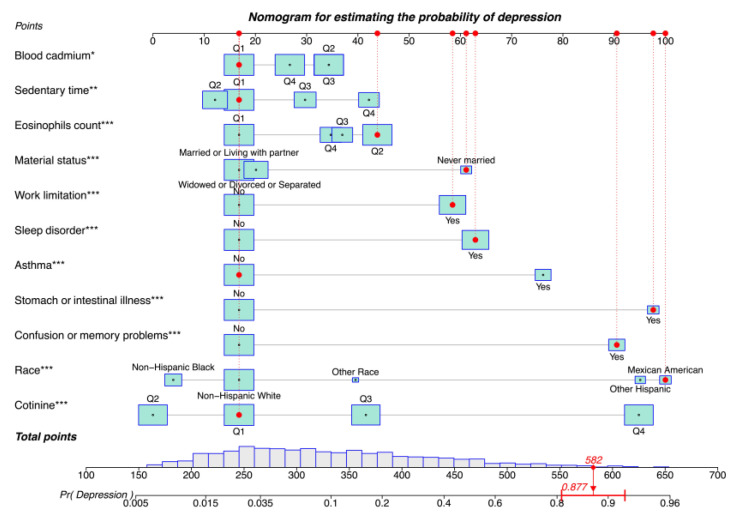
Nomogram for estimating the probability of depression. Asterisks represent statistical significance: * *p* < 0.05, ** *p* < 0.01, and *** *p* < 0.001.

A nomogram model was constructed to predict the risk of depression in patients with CVD. Each predictor was assigned a score from 0 to 100, and a vertical line was drawn downward from the total points to represent the likelihood of depression (Figure 2). Higher scores suggest an increased risk of post-CVD depression. The risk of depression can also be predicted using our web calculator (https://zhaol2022713269.shinyapps.io/DynNomapp/, accessed on 12 March 2025).

Consider the following scenario: Mexican American (scale score of 100), never married (scale score of 61), first quantile blood cadmium (scale score of 17), first quantile sedentary time (scale score of 17), second quantile eosinophil count (scale score of 43), first quantile blood cotinine (scale score of 17), with work limitations (scale score of 58), sleep disorders (scale score of 63), stomach or intestinal illness (scale score of 98), and confusion or memory problems (scale score of 91), but no asthma (scale score of 17). The corresponding scores of the case on the column chart were 100, 61, 17, 17, 43, 17, 58, 63, 98, 91, and 17 points, respectively, with a total score of 582 points, and the corresponding risk probability of depression was 0.877 (87.7%).

**Figure 3:**
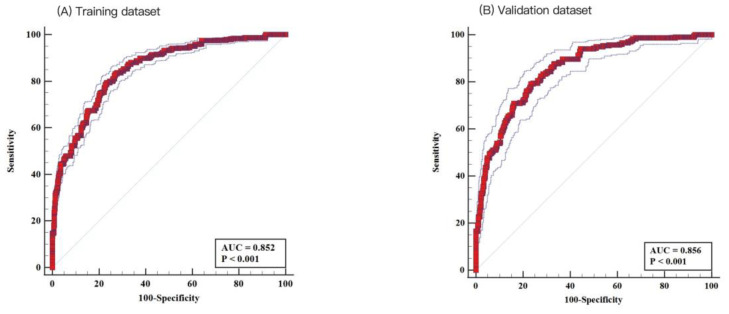
Receiver operating characteristic (ROC) analysis. Nomogram demonstrated good discrimination for depression in patients with cardiovascular disease: (**A**) the training dataset (area under the curve [AUC]: 0.852; 95% confidence interval [CI]: 0.842–0.862) and (**B**) the testing dataset (AUC: 0.856; 95% CI: 0.838–0.872).

The depression risk scoring model had a good discriminative AUC of 0.852 (95% CI = 0.842–0.862, *p* < 0.001) in the training cohort, with a sensitivity of 83.28% and a specificity of 72.95% (Figure 3A). The nomogram model also had a high AUC of 0.856 (95% CI: 0.838–0.872, *p* < 0.001) in the validation cohort, with a sensitivity of 79.14% and specificity of 76.65 (Figure 3B), indicating good discriminative ability.

### 3.6. Performance Assessment of the Risk Prediction Nomogram for Depression

**Figure 4:**
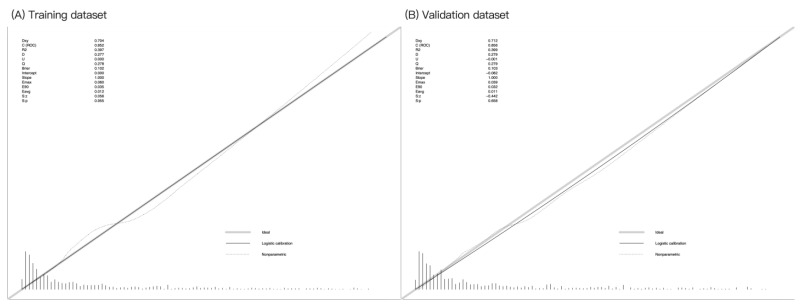
Calibration curves for the training and validation datasets. Calibration plots showing the high accuracy of the absolute risk prediction for depression: (**A**) training dataset (Emax = 0.060, Eavg = 0.012, *p* = 0.955) and (**B**) testing dataset (Emax = 0.039, Eavg = 0.011, *p* = 0.658). If the nomogram has good calibration, a 45-degree diagonal line will be present between the actual depression rate (*y*-axis) and predicted depression probability (*x*-axis). The lack of statistical significance (*p* > 0.05) indicates good calibration, with no difference between the actual and predicted probabilities.

Internal validation using 1000 bootstrap samples showed that the C-index of the nomogram was 0.852 in the training cohort, with a mean absolute error of 0.012 (Figure 4A). The nomogram calibration plot in the training cohort demonstrated acceptable calibration, with a Brier score of 0.102, indicating good calibration with no difference between the actual rate and predicted probabilities. Internal validation using 1000 bootstrap samples showed that the C-index of the nomogram model was 0.863 in the validation cohort, with a mean absolute error of 0.017 (Figure 4B). The nomogram calibration plot demonstrated acceptable calibration, with a Brier score of 0.103, again indicating a close match between the predicted and observed depression rates.

**Figure 5:**
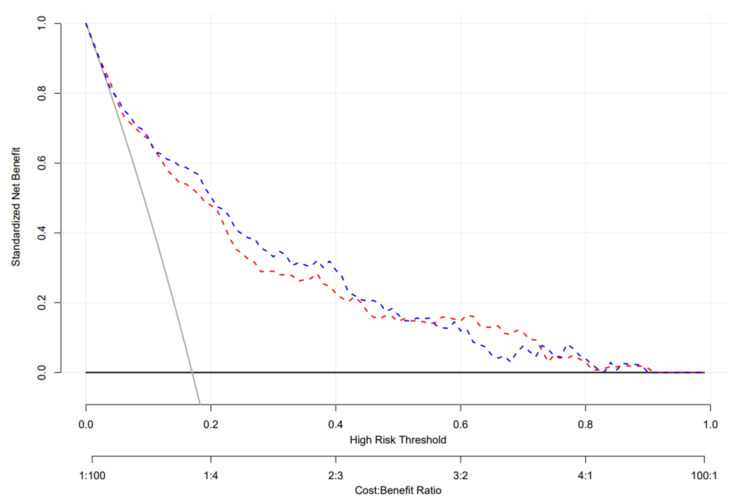
Decision curve analysis of the testing and validation datasets. The decision curve provided greater benefits of the prediction model across the range of 15 to 80%. The blue and red dashed lines indicate the nomograms in the training and validation datasets, respectively; the brown solid line indicates the assumption that all patients are treated, and the dark solid line indicates the assumption that no patient is treated.

DCA was used to evaluate the clinical utility of our nomogram model (Figure 5). The DCA of the training cohort (red dashed line) indicates that the nomogram has a greater net benefit than treated or not treated when the threshold probability ranges from 0.02 to 0.91. The DCA of the validation cohort (blue dashed line) also indicates that the developed nomogram provides a greater net benefit than treatment when the threshold probability falls between 0.05 and 0.90.

**Figure 6:**
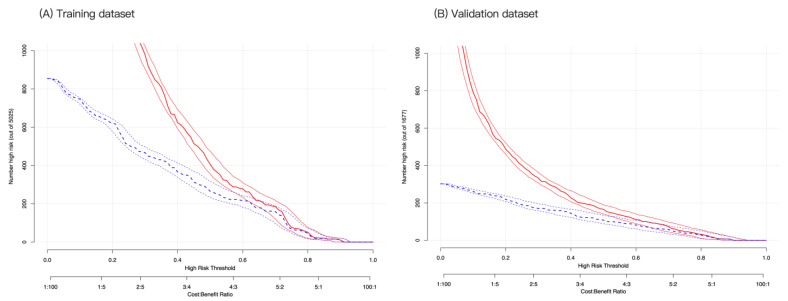
Clinical impact curve analysis for probability stratification of 1000 subjects using a bootstrap random sampling method. At the same horizontal coordinate (high-risk threshold), the vertical coordinates of high-risk patients (blue dashed line) and high-risk patients with depression (red solid line) were compared in the training (**A**) and validation (**B**) cohorts. If the vertical coordinate of the red solid line is higher than that of the blue dashed line, it is considered clinically significant at that point.

Finally, CIC analysis was used for the risk stratification of depression. At a low-risk threshold, the number of depressed populations based on the nomogram model differed from the true depression populations (Figure 6A). At the high-risk threshold, the two CIC curves became more closely aligned with the validation cohort, with a greater degree of coincidence (Figure 6B). Overall, our findings indicate that our nomogram model has greater clinical utility for predicting depression in CVD survivors than intervention or non-intervention.

## 4. Discussion

This population-based cross-sectional study aimed to develop and validate a nomogram for predicting depression risk in patients with CVD. Multivariate regression analysis identified 11 predictors of depression. ROC analysis revealed that our nomogram had a high predictive ability for depression in the datasets used in this study. The calibration curve showed good agreement between the predicted and observed depression probabilities. Decision curve and clinical impact curve analyses showed that our nomogram can be used to predict depression risk in patients with CVD.

Our nomogram model revealed that the risk of depression in patients with CVD varies by race and social and demographic characteristics. In this study, we found that Mexican Americans and other Hispanics were more likely to experience post-CVD depression than non-Hispanic whites. In line with this, a growing body of research shows that Mexican Americans are disproportionately affected by depression disorders [34]. Many risk factors have been linked to mental illness in Mexican Americans, including low socioeconomic status, frequent drinking, health conditions, discrimination, and acculturation [35]. Additionally, those who had never married had a higher risk of developing depression than those who were married, with no difference between those who were married and living with a partner and those living alone. In support of the current findings, living alone (i.e., separated, divorced, widowed, or never married) was a positive risk factor for depression in various populations [36]. There are several possible explanations for this. First, the socio-demographics and lifestyles of Mexican Americans may contribute to increased post-CVD depression risk by activating stress-response pathways (i.e., the sympathetic nervous system and the hypothalamic-pituitary-pituitary-adrenal axis), thereby altering cardiovascular function and structure [37]. Second, psychological stress, such as loneliness and social isolation, may induce unhealthy behavioral changes, such as inactivity, poor diet, smoking, alcoholism, and obesity, as well as increased stress reactivity and exaggerated inflammatory responses, contributing to increased depression and CVD risk [38].

Our findings indicated that post-CVD depression risk is related to health conditions such as stomach or intestinal illness, asthma, and cognitive dysfunction (i.e., confusion and memory problems). In agreement with the current findings, previous studies have linked depression with gastrointestinal symptoms [39] and asthma [40]. Depression may be linked to gastrointestinal issues via the microbiota-gut-brain axis [41], as demonstrated in patients with depression [42]. Pathologically, health conditions can increase the risk of developing depression by triggering anxiety and stress [43], altering brain function and structure [44], or adding medications [45]. Asthma is a psychosomatic disease that causes emotional stress, which can exacerbate symptoms by activating the sympathetic nervous system and the hypothalamic-pituitary-adrenocortical axis [46]. Furthermore, some explanations can be provided for the association between confusion or memory problems and post-CVD depression risk. First, brain structural changes due to hypoperfusion or infarction may contribute to cognitive dysfunction in patients with CVD [47]. Second, impaired cognition in patients with CVD may result from the accumulation of common risk factors such as hypertension, diabetes, dyslipidemia, and atherosclerosis [48]. Third, medications such as anticholinergic drugs prescribed to patients with CVD may induce confusion or memory loss [49].

Our findings indicate that the risk of post-CVD depression is also related to sleep disorders and work limitations. Depressed individuals are more likely to experience sleep disorders than non-depressed individuals [50], implying a strong link between the two mental health conditions. Sleep disturbances, for example, have long been regarded as the most important secondary symptoms of depression. Depression is widely considered a risk factor for insomnia because it disrupts both homeostatic and circadian sleep drives [51]. In addition, sleep deficiency can lead to an inflammatory response that affects peripheral arteries and sympathetic and sensory-motor nerves, leading to blood elevation and increased CVD risk [52]. Depression and CVD impact work limitations. In a retrospective observational study, depression was linked to poor work performance in 100 patients with CVD undergoing a cardiac rehabilitation program [53]. A large, nationally representative survey of the Australian population (*n* = 8841) showed that depression and CVD are synergistically associated with poor work performance [19]. The association between work productivity loss and CVDs has been well-reviewed and summarized in a study [54]. Taken together, the current and previous studies show that depression and CVD have an additive or synergistic effect on patients’ sleep quality and work performance [55].

Our findings suggest that the risk of depression in patients with CVD is linked to sedentary behavior and cotinine. Sedentary behavior is a well-known risk factor for physical and mental health issues. Our findings support the importance of sedentary time and cotinine levels in predicting depression risk in patients with CVD. Sedentarism-related inflammatory responses, such as increased interleukin-6 and decreased brain-derived nerve growth factor levels, are recognized as pathological causes of emotional stress and depression [56]. Cotinine, the primary metabolite of nicotine, has been shown to improve psychiatric symptoms [57] and reduce various consequences of chronic stress, such as reduced working memory and synaptic loss [58]. Given that secondary smoking is a significant risk factor for CVD [59], caution is necessary when considering the association between cotinine and depression.

Metabolomics is a promising tool for understanding the role of heavy metal exposure in the onset and progression of depression [60]. Cadmium is a neurotoxin that can cross the blood-brain barrier and cause oxidative stress, neuro-inflammation, impaired ciliogenesis, and neuronal cell death, resulting in brain damage and mental disorders [61]. We found that the risk of depression was positively related to blood cadmium levels, which supports previous findings on the relationship between blood cadmium and depression in a nationwide sample of young adults [62]. At the same time, blood cadmium levels can be modulated by lifestyle factors such as physical activity. A cross-sectional study involving 5560 adults aged 20 years from the NHANES 2015–2018 showed that the impact of blood cadmium levels on depression risk differed by activity status; the high-intensity activity group had the lowest depression risk, while the inactive group had the highest depression risk [63]. The prognostic role of blood cadmium in depression remains to be confirmed in prospective cohort studies.

Finally, our findings indicate a pathological link between eosinophil counts and depression. Several lines of evidence implicate immune dysfunction in the pathogenesis of depression. Eosinophils are pleiotropic leukocytes that play a role in the initiation and transmission of inflammatory responses, and their elevation is used as a biomarker for mental health conditions like anxiety and depression [64]. A prospective population-based study from Sweden found a potential causal link between duodenal inflammation (mast cells and eosinophils) and psychological distress in functional gut disorders [65]. The relationship between eosinophil count and depression has been thoroughly reviewed and summarized in a scoping review paper of 34 anxiety and depression studies [66].

Taken together, depression is a complex mental illness with far-reaching consequences for an individual’s health. CVD sequelae cause functional impairments, lower the quality of life, and impede reintegration into the family and society. Patients with CVD who do not receive medical care are less likely to return to work than those who do [67]. As a result, CVD has devastating effects on both patients and their families. The risk of depression in patients with CVD is linked to a variety of factors, including inflammatory responses, impaired neuroplasticity, genetic inheritance, the presence of specific metabolites, comorbidities, and unhealthy lifestyles.

This study had some limitations that should be considered. First, several variables used in this study were self-reported, including smoking status, alcohol consumption, sedentary time, and sleep disorders. Therefore, we cannot rule out the possibility of subjective bias influencing the objectivity of the results. Second, the external validation of this study relied on the allocation ratio method, with data sourced solely from the NHANES database. While this approach provides a robust framework for model development and internal validation, it limits the generalizability of the findings to other populations or settings. Ideally, external validation should involve independent datasets from different regions or healthcare systems to confirm the model’s applicability in diverse clinical settings. However, this was not feasible due to differences in data collection practices and available variables across databases. Third, the cross-sectional nature of this study made it impossible to establish a causal relationship between exposure and outcome. Prospective cohort studies are required to clarify these temporal relationships and determine the causal pathways, which would strengthen the predictive validity of the identified risk factors. Third, the cross-sectional nature of this study imposes some limitations. This prevents us from establishing any causal relationships between exposure and outcome because the data were collected at a single time point with no longitudinal follow-up. Furthermore, because this study was based on NHANES data collected between 2007 and 2018, the interpretation and generalizability of the findings are limited by time. These findings reflect associations specific to that period and may not fully capture more recent trends or population shifts. Prospective cohort studies are required to clarify these temporal relationships and identify causal pathways to improve the predictive validity of the identified risk factors. Fourth, our variable selection process used univariate logistic regression to identify predictors before incorporating them into a multivariable model. While this approach reduces noise and improves model interpretability, it has significant limitations. Specifically, univariate screening may exclude variables that are not individually significant but could contribute significantly to the predictive performance when combined with other covariates. This can result in the omission of significant predictors and potentially biased model estimates. Lastly, while the HL test-based calibration of our prediction model provides a good overall fit, it has well-known limitations. In a large sample, the HL test may become overly sensitive to minor deviations or fail to detect significant miscalibrations within specific risk strata. Furthermore, the results may differ depending on how the data are organized, affecting their reliability. As a result, using only the HL test may not provide an accurate picture of model calibration. To address this, we presented calibration plots that visually assessed the agreement between the predicted and observed probabilities across risk levels. The plots show that the model remained well-calibrated across the risk spectrum. Nonetheless, we acknowledge that more comprehensive quantitative measures, such as calibration-in-the-large and calibration slope, would improve the assessment of the model performance.

## 5. Conclusions

Our findings suggest that multiple factors are involved in the risk of depression in patients with CVD. To the best of our knowledge, this is the first study to design and validate a nomogram for predicting depression in patients with CVD. The nomogram model incorporates diverse sets of predictors related to social and demographic factors, comorbidities, health behaviors, metabolites, and exposure to toxic substances. The performance assessment of the nonogram model demonstrated its potential clinical utility for the early screening of depression risk in patients with CVD.

## Figures and Tables

**Table 1:** Descriptive statistics of the study participants by depression status.

Factors	Levels	Without Depression (*n* = 5497)	With Depression (*n* = 1205)	*p*-Value
Age, years (SD)		69.1 (10.6)	59.2 (11.8)	<0.001
Gender, *n* (%)	Female	3352 (60.98)	562 (46.64)	<0.001
	Male	2145 (39.02)	643 (53.36)	
Ethnicity, *n* (%)	Non-Hispanic White	3497 (63.62)	590 (48.96)	<0.001
	Mexican American	444 (8.08)	164 (13.61)	
	Other Hispanic	340 (6.19)	155 (12.86)	
	Non-Hispanic Black	1094 (19.90)	242 (20.08)	
	Others	122 (2.22)	54 (4.48)	
Education, *n* (%)	Less than 9th grade	887 (16.14)	278 (23.07)	<0.001
	9–11th grade	1010 (18.37)	268 (22.24)	
	High school Grad/GED	1476 (26.85)	326 (27.05)	
	Some college or AA degree	1343 (24.43)	307 (25.48)	
	College graduate or above	781 (14.21)	26 (2.16)	
Marital status, *n* (%)	Married/living with a partner	3165 (57.58)	532 (44.15)	<0.001
	Widowed/divorced/separated	2008 (36.53)	494 (41.00)	
	Never married	324 (5.89)	179 (14.85)	
Family income to poverty ratio, *n* (%)	More than 130%	3784 (68.84)	499 (41.41)	<0.001
	Less than 130%	1713 (31.16)	706 (58.59)	
Body mass index, *n* (%)	Underweight	27 (0.49)	9 (0.75)	<0.001
	Normal	919 (16.72)	176 (14.61)	
	Overweight	1765 (32.11)	263 (21.83)	
	Obese	2786 (50.68)	757 (62.82)	
Alcohol status, *n* (%)	Non-drinkers	2210 (40.20)	322 (26.72)	<0.001
	Drinkers	3287 (59.80)	883 (73.28)	
Smoking status, *n* (%)	Never smokers	2022 (36.78)	352 (29.21)	<0.001
	Former smokers	2603 (47.35)	352 (29.21)	
	Current smokers	872 (15.86)	501 (41.58)	
SII levels, *n* (%)	Q1	1340 (24.38)	338 (28.05)	0.001
	Q2	1434 (26.09)	241 (20.00)	
	Q3	1373 (24.98)	306 (25.39)	
	Q4	1350 (24.56)	320 (26.56)	
SIRI levels, *n* (%)	Q1	1278 (23.25)	401 (33.28)	<0.001
	Q2	1408 (25.61)	267 (22.16)	
	Q3	1373 (24.98)	311 (25.81)	
	Q4	1438 (26.16)	226 (18.76)	
Eosinophil count, *n* (%)	Q1	1901 (34.58)	340 (28.22)	<0.001
	Q2	1732 (31.51)	463 (38.42)	
	Q3	923 (16.79)	190 (15.77)	
	Q4	941 (17.12)	212 (17.59)	
Red cell distribution width, *n* (%)	Q1	1516 (27.58)	404 (33.53)	0.001
	Q2	1211 (22.03)	250 (20.75)	
	Q3	1388 (25.25)	292 (24.23)	
	Q4	1382 (25.14)	259 (21.49)	
Blood lead levels, *n* (%)	Q1	1330 (24.20)	407 (33.78)	<0.001
	Q2	1349 (24.54)	266 (22.07)	
	Q3	1380 (25.10)	304 (25.23)	
	Q4	1438 (26.16)	228 (18.92)	
Blood cadmium levels, *n* (%)	Q1	1474 (26.81)	211 (17.51)	<0.001
	Q2	1470 (26.74)	239 (19.83)	
	Q3	1334 (24.27)	310 (25.73)	
	Q4	1219 (22.18)	445 (36.93)	
Blood mercury levels, *n* (%)	Q1	1304 (23.72)	391 (32.45)	<0.001
	Q2	1382 (25.14)	292 (24.23)	
	Q3	1321 (24.03)	336 (27.88)	
	Q4	1490 (27.11)	186 (15.44)	
Blood cotinine levels, *n* (%)	Q1	1537 (27.96)	198 (16.43)	<0.001
	Q2	1493 (27.16)	154 (12.78)	
	Q3	1370 (24.92)	277 (22.99)	
	Q4	1097 (19.96)	576 (47.80)	
Asthma status, *n* (%)	No	4527 (82.35)	668 (55.44)	<0.001
	Yes	970 (17.65)	537 (44.56)	
Osteoarthritis, *n* (%)	No	2175 (39.57)	307 (25.48)	<0.001
	Yes	3322 (60.43)	898 (74.52)	
Stomach or intestinal illness, *n* (%)	No	5000 (90.96)	794 (65.89)	<0.001
	Yes	497 (9.04)	411 (34.11)	
Work limitations, *n* (%)	No	3396 (61.78)	313 (25.98)	<0.001
	Yes	2101 (38.22)	892 (74.02)	
Mobility disorders, *n* (%)	No	3654 (66.47)	655 (54.36)	<0.001
	Yes	1843 (33.53)	550 (45.64)	
Confusion or memory problems, *n* (%)	No	4539 (82.57)	617 (51.20)	<0.001
	Yes	958 (17.43)	588 (48.80)	
Prescription medicine use, *n* (%)	No	61 (1.11)	15 (1.24)	0.802
	Yes	5436 (98.89)	1190 (98.76)	
Sleep duration, *n* (%)	<6 h	713 (12.97)	140 (11.62)	<0.001
	≤8 h	3833 (69.73)	574 (47.63)	
	≤12 h	951 (17.30)	491 (40.75)	
Sleep disorders, *n* (%)	No	3287 (59.80)	356 (29.54)	<0.001
	Yes	2210 (40.20)	849 (70.46)	
Teeth health, *n* (%)	Good	3231 (58.78)	511 (42.41)	<0.001
	Fair	1227 (22.32)	286 (23.73)	
	Poor	1039 (18.90)	408 (33.86)	
Sedentary time levels, *n* (%)	Q1	2035 (37.02)	407 (33.78)	<0.001
	Q2	1440 (26.20)	289 (23.98)	
	Q3	1035 (18.83)	312 (25.89)	
	Q4	987 (17.96)	197 (16.35)	
Physical activity levels, *n* (%)	High	1602 (29.14)	228 (18.92)	<0.001
	Moderate	517 (9.41)	98 (8.13)	
	Low	3378 (61.45)	879 (72.95)	

Body mass index (BMI) was classified as underweight (BMI < 18.5 kg/m^2^), normal weight (BMI 18.5–24.9 kg/m^2^), overweight (BMI 25.0–29.9 kg/m^2^), and obese ( BMI ≥ 30.0 kg/m^2^). Physical activity levels were classified as low (<500 MET-min/week), moderate (500–1000 MET-min/week), or high (>1000 MET-min/week).

**Table 2:** Univariate and multivariate logistic regression analyses of risk factors for depression in patients with cardiovascular disease.

Variables		Univariate Logistic Regression			Multivariable Logistic Regression		
		OR	95% CI	*p*-Value	OR	95% CI	*p*-Value
Age		0.93	0.93–0.94	<0.001	0.97	0.96–0.98	<0.001
Gender							
	Male (reference)						
	Female	1.79	1.58–2.03	<0.001	1.27	1.04–1.55	0.019
Ethnicity							
	Non-Hispanic White (reference)						
	Mexican American	2.19	1.79–2.67	<0.001	3.06	2.29–4.08	<0.001
	Other Hispanic	2.70	2.19–3.33	<0.001	2.57	1.91–3.46	<0.001
	Non-Hispanic Black	1.31	1.11–1.55	0.001	0.6	0.47–0.77	<0.001
	Others	2.62	1.88–3.66	<0.001	1.38	0.89–2.13	0.146
Education attainment							
	<9th Grade (reference)						
	9–11th grade	0.85	0.7–1.02	0.087	0.82	0.63–1.07	0.139
	High school grad/GED	0.70	0.59–0.84	<0.001	0.65	0.49–0.84	0.001
	Some college or AA degree	0.73	0.61–0.88	0.001	0.95	0.72–1.25	0.718
	College graduate or above	0.11	0.07–0.16	<0.001	0.15	0.09–0.26	<0.001
Marital status							
	Married or living with a partner (reference)						
	Widowed or divorced or separated	1.46	1.28–1.67	<0.001	0.92	0.76–1.11	0.365
	Never married	3.29	2.68–4.03	<0.001	1.79	1.33–2.41	<0.001
Family income to poverty ratio							
	≥130% (reference)						
	<130%	3.13	2.75–3.55	<0.001	1.25	1.04–1.52	0.02
Body mass index							
	Underweight (reference)						
	Normal weight	0.57	0.27–1.24	0.159	0.83	0.34–2.02	0.683
	Overweight	0.45	0.21–0.96	0.039	0.65	0.27–1.57	0.34
	Obese	0.82	0.38–1.74	0.597	0.76	0.32–1.81	0.531
Alcohol status							
	Non-drinkers (reference)						
	Drinkers	1.84	1.61–2.12	<0.001	1.27	1.05–1.53	0.015
Smoking status							
	Non-smokers (reference)						
	Former smokers	0.78	0.66–0.91	0.002	0.73	0.59–0.91	0.004
	Current smokers	3.30	2.82–3.86	<0.001	0.53	0.37–0.76	0.001
SII levels							
	Q1 (reference)						
	Q2	0.67	0.56–0.8	<0.001	0.88	0.68–1.13	0.306
	Q3	0.88	0.74–1.05	0.158	1.52	1.14–2.01	0.004
	Q4	0.94	0.79–1.11	0.475	1.32	0.96–1.81	0.09
SIRI levels							
	Q1 (reference)						
	Q2	0.60	0.51–0.72	<0.001	0.44	0.34–0.58	<0.001
	Q3	0.72	0.61–0.85	<0.001	0.51	0.38–0.68	<0.001
	Q4	0.50	0.42–0.6	<0.001	0.48	0.34–0.66	<0.001
Eosinophils count							
	Q1 (reference)						
	Q2	1.49	1.28–1.74	<0.001	1.61	1.31–1.99	<0.001
	Q3	1.15	0.95–1.4	0.156	1.53	1.18–1.99	0.001
	Q4	1.26	1.04–1.52	0.016	1.78	1.38–2.3	<0.001
Red cell distribution width							
	Q1 (reference)						
	Q2	0.77	0.65–0.92	0.004	0.65	0.51–0.82	<0.001
	Q3	0.79	0.67–0.93	0.006	0.85	0.68–1.07	0.163
	Q4	0.70	0.59–0.84	<0.001	0.84	0.66–1.06	0.142
Blood lead levels							
	Q1 (reference)						
	Q2	0.64	0.54–0.77	<0.001	0.68	0.55–0.85	0.001
	Q3	0.72	0.61–0.85	<0.001	0.83	0.66–1.05	0.121
	Q4	0.52	0.43–0.62	<0.001	0.64	0.49–0.84	0.001
Blood cadmium levels							
	Q1 (reference)						
	Q2	1.14	0.93–1.39	0.209	2.02	1.55–2.63	<0.001
	Q3	1.62	1.34–1.96	<0.001	1.81	1.38–2.39	<0.001
	Q4	2.55	2.13–3.05	<0.001	1.82	1.33–2.48	<0.001
Blood mercury levels							
	Q1 (reference)						
	Q2	0.70	0.59–0.83	<0.001	0.67	0.54–0.85	0.001
	Q3	0.85	0.72–1	0.05	1.07	0.85–1.34	0.569
	Q4	0.42	0.34–0.5	<0.001	0.78	0.6–1.02	0.066
Blood cotinine levels							
	Q1 (reference)						
	Q2	0.80	0.64–1	0.05	0.66	0.5–0.87	0.003
	Q3	1.57	1.29–1.91	<0.001	1.03	0.79–1.35	0.814
	Q4	4.08	3.41–4.88	<0.001	3.26	2.25–4.71	<0.001
Asthma?							
	No (reference)						
	Yes	3.75	3.28–4.29	<0.001	1.87	1.54–2.27	<0.001
Osteoarthritis?							
	No (reference)						
	Yes	1.92	1.66–2.2	<0.001	1.31	1.08–1.58	0.006
Stomach or intestinal illness?							
	No (reference)						
	Yes	5.21	4.48–6.05	<0.001	4.14	3.36–5.09	<0.001
Work limitation?							
	No (reference)						
	Yes	4.61	4.01–5.3	<0.001	1.48	1.22–1.79	<0.001
Mobility disorders?							
	No (reference)						
	Yes	1.66	1.47–1.89	<0.001	0.92	0.76–1.11	0.367
Confusion or memory problems?							
	No (reference)						
	Yes	4.52	3.95–5.16	<0.001	3.29	2.72–3.96	<0.001
Prescription medicine use?							
	No (reference)						
	Yes	0.89	0.5–1.57	0.688	NA	NA	NA
Sleep duration							
	8–12 h (reference)						
	6–8 h	0.76	0.62–0.93	0.008	0.85	0.65–1.12	0.258
	less than 6 h	2.63	2.13–3.25	<0.001	1.59	1.18–2.14	0.002
Sleep disorders							
	No (reference)						
	Yes	3.55	3.1–4.06	<0.001	1.76	1.47–2.12	<0.001
Teeth health conditions							
	Good (reference)						
	Fair	1.47	1.26–1.73	<0.001	0.9	0.73–1.11	0.329
	Poor	2.48	2.14–2.88	<0.001	1.02	0.84–1.25	0.843
Sedentary time							
	Q1 (reference)						
	Q2	1.0	0.85–1.18	0.967	1.06	0.85–1.32	0.617
	Q3	1.51	1.28–1.78	<0.001	1.54	1.21–1.96	<0.001
	Q4	1.0	0.83–1.2	0.983	1.7	1.31–2.22	<0.001
Physical activity levels							
	High (reference)						
	Moderate	1.33	1.03–1.72	0.029	1.27	0.88–1.83	0.209
	Low	1.83	1.56–2.14	<0.001	1.33	1.07–1.66	0.011

GED, gender equivalency diploma; SII, systemic immune-inflammation index; SIRI, systemic inflammatory response index. Body mass index (BMI) was classified as underweight (BMI < 18.5 kg/m^2^), normal weight (BMI 18.5–24.9 kg/m^2^), overweight (BMI 25.0–29.9 kg/m^2^), and obese (BMI ≥ 30.0 kg/m^2^). Physical activity was classified as low (<500 MET-min/week), moderate (500–1000 MET-min/week), or high (>1000 MET-min/week).

## Data Availability

The datasets generated and/or analyzed during the current study are available in the NHANES [https://www.cdc.gov/nchs/nhanes/about/index.html/, accessed on 10 September 2024].

## Abbreviations

The following abbreviations are used in this manuscript:

CVD	Cardiovascular disease
AUC	Area under the curve
WHO	World Health Organization
NCHS	National Center for Health Statistics
CDC	Center for Disease Control and Prevention
NHNES	National Health and Nutrition Examination Survey
BIC	Bayesian Information Criterion
ROCAUC	Area the under the receiver operating characteristic curve
PRAUC	Area under the precision-recall curve
MCQ	Medical conditions questionnaire
PHQ-9	Nine-item Patient Health Questionnaire
DSM-5	Diagnostic and Statistical Manual of Mental Disorders, firth edition
PA	Physical activity
BMI	Body mass index
LPA	Light physical activity
MPA	Moderate physical activity
VPA	Vigorous physical activity
METs	Metabolic equivalent tasks
CBC	Complete blood count
SII	Systemic immune-inflammation index
SIRI	Systemic inflammatory response index
VIF	Variance inflation factor
OR	Odds ratio
CI	Confidence interval
DCA	Decision curve analysis
CIC	Clinical impact curve
HL	Hosmer-Lemeshow

## Appendix A

**Table A1:** Descriptive statistics of the study participants by depression status in each cohort.

Factors	Levels	Training Cohort (*n* = 5025)		*p*-Value	Validation Cohort (*n* = 1677)		*p*-Value
		Not Depressed	Depressed		Not Depressed	Depressed	
		(*n* = 4122)	(*n* = 903)		(*n* = 1375)	(*n* = 302)	
Age (mean (SD))		69.04 (10.54)	59.16 (11.68)	<0.0001	69.25 (10.77)	59.10 (12.27)	<0.0001
Gender (%)	Male	2519 (61.11)	424 (46.95)	<0.0001	833 (60.58)	138 (45.70)	<0.0001
	Female	1603 (38.89)	479 (53.05)		542 (39.42)	164 (54.30)	
Race (%)	Non-Hispanic White	2618 (63.51)	444 (49.17)	<0.0001	879 (63.93)	146 (48.34)	<0.0001
	Mexican American	325 (7.88)	122 (13.51)		119 (8.65)	42 (13.91)	
	Other Hispanic	261 (6.33)	116 (12.85)		79 (5.75)	39 (12.91)	
	Non-Hispanic Black	823 (19.97)	179 (19.82)		271 (19.71)	63 (20.86)	
	Other Race	95 (2.30)	42 (4.65)		27 (1.96)	12 (3.97)	
Education (%)	Less Than 9th Grade	655 (15.89)	218 (24.14)	<0.0001	232 (16.87)	60 (19.87)	<0.0001
	9-11th Grade	751 (18.22)	196 (21.71)		259 (18.84)	72 (23.84)	
	High School Grad/GED	1112 (26.98)	244 (27.02)		364 (26.47)	82 (27.15)	
	Some College or AA degree	1012 (24.55)	224 (24.81)		331 (24.07)	83 (27.48)	
	College Graduate or above	592 (14.36)	21 (2.33)		189 (13.75)	5 (1.66)	
Material status (%)	Married/Living with partner	2392 (58.03)	393 (43.52)	<0.0001	773 (56.22)	139 (46.03)	<0.0001
	Widowed/Divorced/Separated	1488 (36.10)	375 (41.53)		520 (37.82)	119 (39.40)	
	Never married	242 (5.87)	135 (14.95)		82 (5.96)	44 (14.57)	
Family income to poverty ratio (%)	More than 130%	2829 (68.63)	364 (40.31)	<0.0001	955 (69.45)	135 (44.70)	<0.0001
	Less than 130%	1293 (31.37)	539 (59.69)		420 (30.55)	167 (55.30)	
BMI (%)	Underweight (<18.5)	19 (0.46)	5 (0.55)	<0.0001	8 (0.58)	4 (1.32)	0.0017
	Normal (18.5 to <25)	690 (16.74)	128 (14.17)		229 (16.65)	48 (15.89)	
	Overweight (25 to <30)	1316 (31.93)	195 (21.59)		449 (32.65)	68 (22.52)	
	Obese (30 or greater)	2097 (50.87)	575 (63.68)		689 (50.11)	182 (60.26)	
Alcohol status (%)	Non-drinker	1637 (39.71)	243 (26.91)	<0.0001	573 (41.67)	79 (26.16)	<0.0001
	Drinker	2485 (60.29)	660 (73.09)		802 (58.33)	223 (73.84)	
Smoking status (%)	Never smoker	1524 (36.97)	266 (29.46)	<0.0001	498 (36.22)	86 (28.48)	<0.0001
	Former smoker	1930 (46.82)	263 (29.13)		673 (48.95)	89 (29.47)	
	Current smoker	668 (16.21)	374 (41.42)		204 (14.84)	127 (42.05)	
SII (%)	Q1	1004 (24.36)	266 (29.46)	0.0001	336 (24.44)	72 (23.84)	0.2379
	Q2	1106 (26.83)	184 (20.38)		328 (23.85)	57 (18.87)	
	Q3	1010 (24.50)	216 (23.92)		363 (26.40)	90 (29.80)	
	Q4	1002 (24.31)	237 (26.25)		348 (25.31)	83 (27.48)	
SIRI (%)	Q1	977 (23.70)	300 (33.22)	<0.0001	301 (21.89)	101 (33.44)	0.0002
	Q2	1055 (25.59)	209 (23.15)		353 (25.67)	58 (19.21)	
	Q3	1016 (24.65)	237 (26.25)		357 (25.96)	74 (24.50)	
	Q4	1074 (26.06)	157 (17.39)		364 (26.47)	69 (22.85)	
Eosinophils count (%)	Q1	1440 (34.93)	252 (27.91)	0.0001	461 (33.53)	88 (29.14)	0.0318
	Q2	1307 (31.71)	343 (37.98)		425 (30.91)	120 (39.74)	
	Q3	674 (16.35)	143 (15.84)		249 (18.11)	47 (15.56)	
	Q4	701 (17.01)	165 (18.27)		240 (17.45)	47 (15.56)	
Red cell distribution width (%)	Q1	1135 (27.54)	305 (33.78)	0.0024	381 (27.71)	99 (32.78)	0.1175
	Q2	917 (22.25)	186 (20.60)		294 (21.38)	64 (21.19)	
	Q3	1048 (25.42)	214 (23.70)		340 (24.73)	78 (25.83)	
	Q4	1022 (24.79)	198 (21.93)		360 (26.18)	61 (20.20)	
Blood lead (%)	Q1	970 (23.53)	301 (33.33)	<0.0001	360 (26.18)	106 (35.10)	0.0162
	Q2	1026 (24.89)	203 (22.48)		323 (23.49)	63 (20.86)	
	Q3	1029 (24.96)	232 (25.69)		351 (25.53)	72 (23.84)	
	Q4	1097 (26.61)	167 (18.49)		341 (24.80)	61 (20.20)	
Blood cadmium (%)	Q1	1103 (26.76)	165 (18.27)	<0.0001	371 (26.98)	46 (15.23)	<0.0001
	Q2	1107 (26.86)	172 (19.05)		363 (26.40)	67 (22.19)	
	Q3	974 (23.63)	232 (25.69)		360 (26.18)	78 (25.83)	
	Q4	938 (22.76)	334 (36.99)		281 (20.44)	111 (36.75)	
Mercury (%)	Q1	992 (24.07)	307 (34.00)	<0.0001	312 (22.69)	84 (27.81)	0.0020
	Q2	1035 (25.11)	210 (23.26)		347 (25.24)	82 (27.15)	
	Q3	975 (23.65)	250 (27.69)		346 (25.16)	86 (28.48)	
	Q4	1120 (27.17)	136 (15.06)		370 (26.91)	50 (16.56)	
Cotinine (%)	Q1	1137 (27.58)	150 (16.61)	<0.0001	400 (29.09)	48 (15.89)	<0.0001
	Q2	1134 (27.51)	110 (12.18)		359 (26.11)	44 (14.57)	
	Q3	1020 (24.75)	211 (23.37)		350 (25.45)	66 (21.85)	
	Q4	831 (20.16)	432 (47.84)		266 (19.35)	144 (47.68)	
Asthma (%)	No	3379 (81.97)	503 (55.70)	<0.0001	1148 (83.49)	165 (54.64)	<0.0001
	Yes	743 (18.03)	400 (44.30)		227 (16.51)	137 (45.36)	
Osteoarthritis (%)	No	1626 (39.45)	225 (24.92)	<0.0001	549 (39.93)	82 (27.15)	<0.0001
	Yes	2496 (60.55)	678 (75.08)		826 (60.07)	220 (72.85)	
Stomach or intestinal illness (%)	No	3757 (91.15)	596 (66.00)	<0.0001	1243 (90.40)	198 (65.56)	<0.0001
	Yes	365 (8.85)	307 (34.00)		132 (9.60)	104 (34.44)	
Work limitation (%)	No	2540 (61.62)	238 (26.36)	<0.0001	856 (62.25)	75 (24.83)	<0.0001
	Yes	1582 (38.38)	665 (73.64)		519 (37.75)	227 (75.17)	
Mobility disorder (%)	No	2737 (66.40)	488 (54.04)	<0.0001	917 (66.69)	167 (55.30)	0.0002
	Yes	1385 (33.60)	415 (45.96)		458 (33.31)	135 (44.70)	
Confusion memory problems (%)	No	3380 (82.00)	457 (50.61)	<0.0001	1159 (84.29)	160 (52.98)	<0.0001
	Yes	742 (18.00)	446 (49.39)		216 (15.71)	142 (47.02)	
Prescribed medicine use (%)	No	54 (1.31)	10 (1.11)	0.7429	7 (0.51)	5 (1.66)	0.0778
	Yes	4068 (98.69)	893 (98.89)		1368 (99.49)	297 (98.34)	
Sleep duration (%)	<6	534 (12.95)	104 (11.52)	<0.0001	179 (13.02)	36 (11.92)	<0.0001
	≤8	2864 (69.48)	435 (48.17)		969 (70.47)	139 (46.03)	
	≤12	724 (17.56)	364 (40.31)		227 (16.51)	127 (42.05)	
Sleep disorder (%)	No	2479 (60.14)	276 (30.56)	<0.0001	808 (58.76)	80 (26.49)	<0.0001
	Yes	1643 (39.86)	627 (69.44)		567 (41.24)	222 (73.51)	
Teeth health (%)	Good	2423 (58.78)	376 (41.64)	<0.0001	808 (58.76)	135 (44.70)	<0.0001
	Fair	924 (22.42)	215 (23.81)		303 (22.04)	71 (23.51)	
	Poor	775 (18.80)	312 (34.55)		264 (19.20)	96 (31.79)	
Sedentary time (%)	Q1	1534 (37.21)	307 (34.00)	<0.0001	501 (36.44)	100 (33.11)	0.0505
	Q2	1089 (26.42)	211 (23.37)		351 (25.53)	78 (25.83)	
	Q3	762 (18.49)	232 (25.69)		273 (19.85)	80 (26.49)	
	Q4	737 (17.88)	153 (16.94)		250 (18.18)	44 (14.57)	
Physical activity (%)	Vigorous	1212 (29.40)	185 (20.49)	<0.0001	390 (28.36)	43 (14.24)	<0.0001
	Moderate	390 (9.46)	76 (8.42)		127 (9.24)	22 (7.28)	
	Low	2520 (61.14)	642 (71.10)		858 (62.40)	237 (78.48)	

BMI, body mass index; SII, systemic immune-inflammation index; SIRI, systemic inflammatory response index.

**Table A2:** The VIF values of the selected variables.

Variable	lambda.1se
Race	1.310458
Material status	2.293593
Blood cadmium	3.727409
Cotinine	2.470422
Asthma	1.367038
Stomach intestinal illness	1.179397
Confusion memory problems	1.753833
Sleep disorder	2.169977
Eosinophils count	2.537814
Walk limitation	1.673927
Sedentary time	3.124892
